# Rough gold films as broadband absorbers for plasmonic enhancement of TiO_2_ photocurrent over 400–800 nm

**DOI:** 10.1038/srep33049

**Published:** 2016-09-09

**Authors:** Furui Tan, Tenghao Li, Ning Wang, Sin Ki Lai, Chi Chung Tsoi, Weixing Yu, Xuming Zhang

**Affiliations:** 1 Department of Applied Physics, The Hong Kong Polytechnic University, Hong Kong, P. R. China; 2 The Hong Kong Polytechnic University Shenzhen Research Institute, Shenzhen, P. R. China; 3 Key Laboratory of Spectral Imaging Technology, Xi’an Institute of Optics and Precision Mechanics, Chinese Academy of Sciences, Xi’an, P. R. China

## Abstract

Recent years have witnessed an increasing interest in highly-efficient absorbers of visible light for the conversion of solar energy into electrochemical energy. This study presents a TiO_2_-Au bilayer that consists of a rough Au film under a TiO_2_ film, which aims to enhance the photocurrent of TiO_2_ over the whole visible region and may be the first attempt to use rough Au films to sensitize TiO_2_. Experiments show that the bilayer structure gives the optimal optical and photoelectrochemical performance when the TiO_2_ layer is 30 nm thick and the Au film is 100 nm, measuring the absorption 80–90% over 400–800 nm and the photocurrent intensity of 15 μA·cm^−2^, much better than those of the TiO_2_-AuNP hybrid (i.e., Au nanoparticle covered by the TiO_2_ film) and the bare TiO_2_ film. The superior properties of the TiO_2_-Au bilayer can be attributed to the rough Au film as the plasmonic visible-light sensitizer and the photoactive TiO_2_ film as the electron accepter. As the Au film is fully covered by the TiO_2_ film, the TiO_2_-Au bilayer avoids the photocorrosion and leakage of Au materials and is expected to be stable for long-term operation, making it an excellent photoelectrode for the conversion of solar energy into electrochemical energy in the applications of water splitting, photocatalysis and photosynthesis.

TiO_2_ has been extensively investigated as a photoelectrode for various applications, including photoelectrochemical water splitting^1^,^2^, pollutant degradation^3^,^4^, *et al*., thanks to its excellent chemical stability, photocorrosion resistance and low cost. As an n-type semiconductor with a wide band gap around 3.2 eV, it can only absorb UV light (cutoff wavelength ~388 nm), causing a low utilization efficiency of solar light that spans from UV to infrared region. Therefore, an ideal photoelectrode for the solar photocatalysis should have a broadband absorption in the wavelength range of 400–800 nm. For practical applications, the photoelectrode should be stable, low cost and easy fabrication of large-area samples. In the past decade, plasmonic metal nanoparticles (NPs) have been widely used to sensitize the host semiconductor TiO_2_ to visible light^5^,^6^. However, most studies rely on the metal NPs with uniform shape and size to obtain visible light response, whose absorption due to the localized surface plasmon resonance (LSPR) is often limited to a narrow spectral range^7^,^8^. Since the broad absorption band can be regarded as a combination of many absorption peaks, Au NPs with different sizes and/or shapes may be used, but this is not favorable due to the fabrication complexity. Additionally, multiple resonant peaks could also be obtained by use of the multipolar resonances in different directions^9^,^10^, but the processing techniques appear to be complicated^11^,^12^,^13^. To address this problem, broadband resonant nanostructures with simple fabrication are highly desired.

Here we present an original design of plasmonic strong absorber for solar energy harvesting and photocatalysis application in the visible region. This absorber, the TiO_2_-Au bilayer, consists of two simple layers, a rough Au film at the bottom as the plasmonic antenna to absorb optical energy for photo-excited electron generation and a TiO_2_ film at the top as the photocatalyst to receive the electrons for the photocurrent transfer. Simple processes like the magnetic sputtering and the thermal annealing are adopted to fabricate the rough Au film, which is equivalent to a collection of random Au nanostructures with different geometries and sizes. Consequentially, this TiO_2_-Au bilayer supports the plasmon resonance of individual nanostructures and the plasmonic coupling of neighboring nanostructures, and enables the broadband absorption in the visible wavelength range from 400 to 800 nm, acting effectively as a broadband visible-light absorber. This kind of visible-light absorbers have already been used in photothermal systems that rely on the heat^10^,^14^, but their great potential for the photocurrent generation has been merely overlooked. The photoelectrochemical performance of the TiO_2_-Au bilayer demonstrates apparent enhancement as compared to the TiO_2_-AuNP hybrid (i.e., Au NPs covered by TiO_2_ film) and the bare TiO_2_ film, indicating superior ability of electron generation and transfer. We further examine the influence of the thickness of TiO_2_ film on the properties of the TiO_2_-Au bilayer, and find that the optimal thickness is 30 nm. The fabrication techniques of the TiO_2_-Au bilayer are relatively simple and cost-effective. Additionally, in contrast to the previously reported AuNP-TiO_2_ systems (i.e., Au NPs on top of the TiO_2_ film)^15^,^16^,^17^, the TiO_2_-Au bilayer buries the Au film under the TiO_2_ film and thus protects the Au film from the photocorrosion and leakage during the photochemical reactions, making it promising for long-term operation.

## Results

### Comparison of TiO_2_-Au bilayer, TiO_2_-AuNP hybrid and bare TiO_2_ film

**Figure 1:**
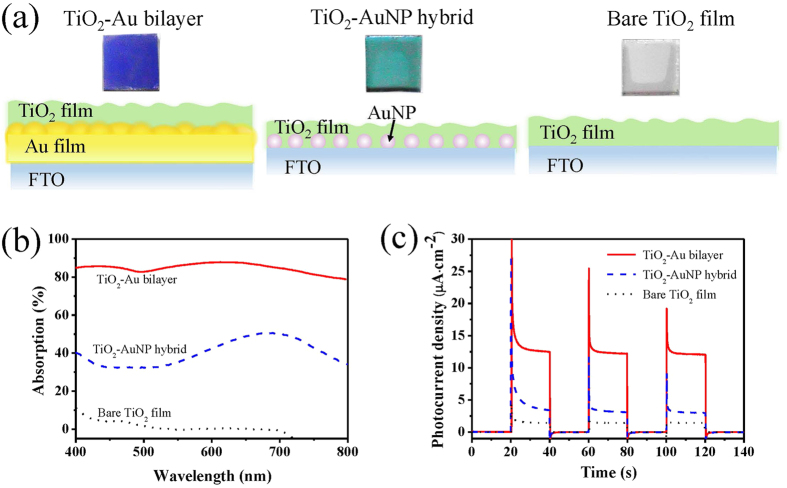
(**a**) Photographs and schematic diagrams of the three samples, from left to right, the TiO_2_-Au bilayer, the TiO_2_-AuNP hybrid and the bare TiO_2_ film, each sample has the footprint of 1 cm × 1 cm; (**b**) the absorption spectra and (**c**) *I-t* plots of the TiO_2_-Au bilayer (red solid lines), the TiO_2_-AuNP hybrid (blue dashed lines) and the bare TiO_2_ film (black dotted lines), respectively. The TiO_2_ films of the three samples are all 30 nm thick. The bias potential for the *I-t* measurement is 0 V vs SCE (i.e., saturated calomel electrode).

Figure 1a shows the photos and the schematic diagrams of the proposed structures, including the TiO_2_-Au bilayer, the TiO_2_-AuNP hybrid and the bare TiO_2_ film. For the bare Au film deposited on the FTO glass, it appears dark yellow. After this bare Au film is covered by the 30-nm TiO_2_ film to form the TiO_2_-Au bilayer, it turns to dark blue after the annealing treatment (see the left of Fig. 1a). The characteristic color of Au NPs is pinkish, which turns into jade color after the coverage of the 30-nm-thick TiO_2_ film to form the TiO_2_-AuNP hybrid (see the middle of Fig. 1a). The bare TiO_2_ film exhibits a light gray color (see the right of Fig. 1a). It is noted that the edge parts of samples have slightly different colors from the central parts, because the central parts have TiO_2_ deposited only on one side, whereas the edge parts are covered on both sides due to some process details. One-sided TiO_2_ deposition is favorable for optical spectrum measurements as compared to two-sided TiO_2_ deposition since the latter may result in a nonuniform film on the back side. In experiments, only the central parts with a diameter of 1 mm is used to investigate the photoelectrochemical properties^18^.

A key step to improving the photocurrent is to increase the optical absorption in visible range. Figure 1b compares the absorption spectra of the TiO_2_-Au bilayer, the TiO_2_-AuNP hybrid and the bare TiO_2_ film. Here the absorption *A* is calculated by the equation *A* = 1 − *R* − *T*, where *R* and *T* are the normalized reflection and transmission, respectively, as shown in Fig. S2a,b. The bare TiO_2_ film shows high reflection and transmission over 400–800 nm, resulting in very weak absorption (see Fig. 1b). The TiO_2_-AuNP hybrid shows obvious enhancement of absorption, especially near 680 nm, which can be attributed to the LSPR effect of the Au NPs. In contrast, the TiO_2_-Au bilayer exhibits much stronger absorption. Particularly, the absorption spectrum is flat over the whole range of 400–800 nm, with the minimum absorption of 80% and the maximum 90%.

The light-harvesting capability plays an important role for the enhanced photoelectrochemical activity under the visible light irradiation. The photoactivities of these three TiO_2_-based samples are evaluated using the *I-t* technique. Figure 1c plots the curves of photocurrent density at the 0-V bias potential. The transient photocurrent responses of the three TiO_2_-based samples are measured for several on-off cycles of irradiation. The rise and fall of the photocurrent respond well to the switching on and off of the visible-light irradiation (wavelength >400 nm). The photocurrent drops to zero as soon as the irradiation is turned off, and recovers immediately when the irradiation is turned on again. It indicates that the current is completely due to the visible-light response of the photoelectrode, and the charge transport is very fast. As can be read from Fig. 1c, the TiO_2_-Au bilayer produces the transient photocurrent density of 12.4 μA·cm^−2^, higher than the TiO_2_-AuNP hybrid (3.2 μA·cm^−2^) and the bare TiO_2_ film (1.5 μA·cm^−2^) by a factor of 3.8 and 8.2. This indicates that the TiO_2_-Au bilayer has the best effect on charge generation, separation and transport^19^. In addition, the order of the photocurrent densities agrees with the absorption measurements in Fig. 1b. As a supplement, the *I-t* plots of the TiO_2_-AuNP hybrid and the bare TiO_2_ film are shown in Fig. S3(c,d), under monochromatic lights at the wavelengths of 420, 450, 475, 500, 520, 550, 600 and 650 nm, respectively. For quantitative comparison, the action spectra (i.e., photocurrent density versus light wavelength) are plotted in Fig. S3e for the three samples. It is seen that the TiO_2_-Au bilayer has the highest response under every monochromatic light illumination, showing significant enhancement by a factor of about 2 as compared to the TiO_2_-AuNP hybrid and the bare TiO_2_ film.

For the TiO_2_-Au bilayer and the TiO_2_-AuNP hybrid, their differences in *I-t* plot and action spectrum can be explained below. From the physical mechanisms, the enhancement of photocurrent in the TiO_2_-Au bilayer and the TiO_2_-AuNP hybrid should be attributed to the Au nanostructures and the formation of Schottky junctions at the interfaces of Au and TiO_2_. The former enables the optical absorption to visible light, whereas the latter improves the separation of photo-generated electron-hole pairs and suppresses the recombination of photogenerated charge. But there are still some differences between the TiO_2_-Au bilayer and the TiO_2_-AuNP hybrid, lying in mostly the density of Au nanostructures. In the TiO_2_-Au bilayer, the rough Au film can be regarded as an assembly of densely-packed random Au nanostructures (e.g., NPs, nanocavities) with different shapes and sizes (see detailed discussion below). The dense packing results in a large number of Au nanostructures, and the LSPR effect of every single Au nanostructure sums up to high absorption over a wide span of wavelength; moreover, the dense packing enables the plasmonic coupling of neighboring Au nanostructures and further enhances the optical absorption. In addition, the dense packing provides good electrical connections among the Au nanostructures for the transport and distribution of photogenerated charges. Furthermore, the rough Au film provides large interface area between the Au and TiO_2_ for the Schottky junction and enables the efficient transfer of photo-excited electrons and holes between Au to TiO_2_. In comparison, the TiO_2_-AuNP hybrid has relatively low density of Au NPs, causing not that ideal absorption and charge transfer. As a result, the TiO_2_-Au bilayer has stronger absorption than the TiO_2_-AuNP hybrid and is expected to have larger photocurrent.

**Figure 2:**
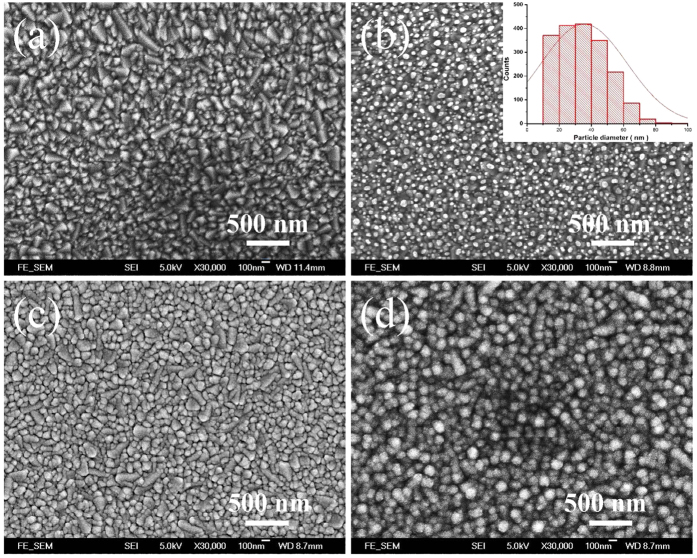
Scanning electron micrographs of the surface morphologies of different layers of the samples. (**a**) FTO glass; (**b**) Au NPs on FTO glass, deposited by the sputtering process; (**c**) rough Au film on FTO glass; (**d**) ALD-deposited TiO_2_ film on the rough Au film. The inset in (**b**) is the histogram of the size of Au NPs.

Figure 2 displays the surface morphologies of the respective layers of the three types of TiO_2_-based samples. The surface of FTO glass itself is already very rough as shown in Fig. 2a. For the Au NPs on the FTO glass, the deposited thin Au film with a thickness around 8 nm is transformed to discontinuous Au NPs after the annealing as shown in Fig. 2b 20,21. When viewed from the top, the Au NPs exhibit nearly round shape and similar particle size. The histogram of the particle size distribution is obtained with the free software ImageJ as shown in the inset of Fig. 2b. The Au NPs have an average size of 35 nm with a standard deviation of approximately 11 nm. When the thickness of the deposited Au film is increased to 100 nm, the surface of Au film becomes rough and thus a pattern of densely packed metallic cluster grows up, which seems like a collection of many Au nanostructures with large variations of shape and size, as shown in Fig. 2c. The TiO_2_ film deposited by the atomic layer deposition (ALD) process on the rough Au film has quite uniform grain size of TiO_2_ nanoparticles as shown in Fig. 2d. These structural layers in a larger area are shown in Fig. S1.

### Influence of TiO_2_ film thickness of TiO_2_-Au bilayer

**Figure 3:**
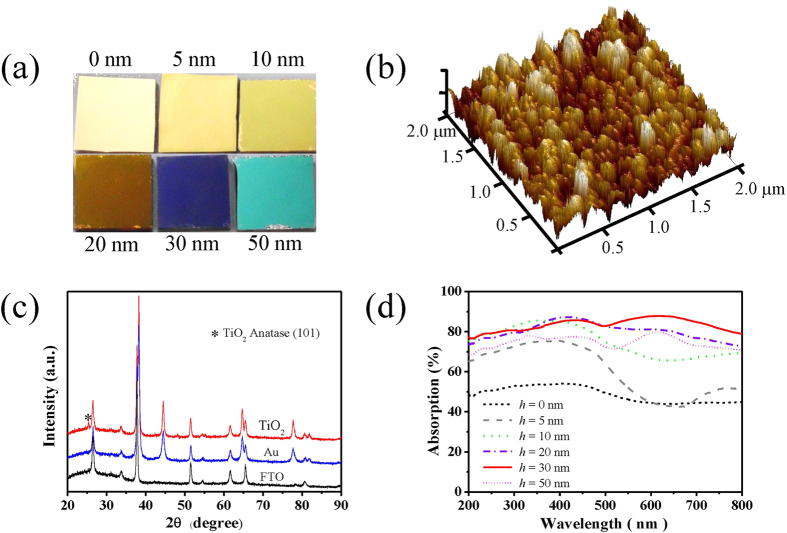
(**a**) Photographs of the TiO_2_-Au bilayer samples with the TiO_2_ thicknesses of 0, 5, 10, 20, 30 and 50 nm. The films are deposited by the ALD at 100 °C and then annealed at 500 °C for 1 h; (**b**) atomic force micrograph of the surface morphology of the rough Au film on the FTO substrate; (**c**) the XRD patterns of the bare TiO_2_ film, the bare Au film and the FTO substrate; and (**d**) the absorption spectra of different TiO_2_-Au bilayer samples.

The thickness of the TiO_2_ film plays an important role in the optical and photoelectrochemical properties of the TiO_2_-Au bilayer. To examine this influence, a series of TiO_2_ thin film are deposited onto the Au film by the ALD method, including 0, 5, 10, 20, 30 and 50 nm. The ALD is a precision growth technique that can passivate surface states to decrease the surface recombination velocity, which can synthesize thin film from only a few atomic layers to hundreds of nanometers^22^,^23^. Its layer-by-layer deposition allows highly conformal coating even on the dense and rough surfaces of nanostructures. Therefore, the as-deposited TiO_2_ films with excellent step coverage have amorphous structure. The thickness of TiO_2_ film can be controlled by the deposition time according to the deposition condition. Color photographs of the TiO_2_-Au bilayer samples with various TiO_2_ thicknesses are shown in Fig. 3a. The sample with the 0-nm TiO_2_ film represents only the rough Au film, which looks bright yellow. The samples appear deepened yellow when the TiO_2_ thickness is increased from 5 to 20 nm. The sample becomes dark blue when the TiO_2_ thickness is 30 nm, and turns to jade for the 50-nm TiO_2_. The atomic force microscopy (AFM) is also employed to characterize the surface morphology of the rough Au film (Fig. 3b), which agrees well with the measured SEM image (Fig. 2c). The mean roughness of the rough Au film is approximately 5 nm within the area of 2 μm × 2 μm and the root-mean-square (rms) roughness is 15 nm, indicating significant surface roughening. Moreover, the XRD patterns (Fig. 3c) of the TiO_2_ films match the Joint Committee on Powder Diffraction Standards File (JCPDS no. 21-1272), which confirms the anatase phase of TiO_2_^24^. It is well known that the anatase phase of TiO_2_ has greater photocatalytic activity than the rutile phase due to the lower conduction band and higher hydrophilicity^25^. For the influence of the Au film thickness, it has little effect as long as the Au film is thick enough to block all the light and rough enough to enable strong, broadband absorption of visible light. Therefore, this study does not investigate the influence of Au film thickness, but simply maintains the Au film thickness at 100 nm, which is much thicker than the skin depth of Au film (about 13 nm). The surface of the 100-nm Au film after annealing is still very rough for plasmonic absorption^26^.

The absorption spectra of these TiO_2_-Au bilayer samples with different TiO_2_ thicknesses are also determined by *A* = 1 − *R* − *T* as shown in Fig. 3d. It is seen that the absorption increases with the TiO_2_ thickness from 0 to 30 nm, but starts to decrease when the thickness is over 30 nm. More details of the reflection and transmission spectra are given in Fig. S2 of the Supplementary Information. Figure S2c shows that the reflection first decreases with thicker TiO_2_, reaches the minimum at 30 nm and then increases. From Fig. S2d, the transmission shares the similar trend, except that it has two peaks at around 500 nm and 700 nm, though the level of transmission is always <10%.

**Figure 4:**
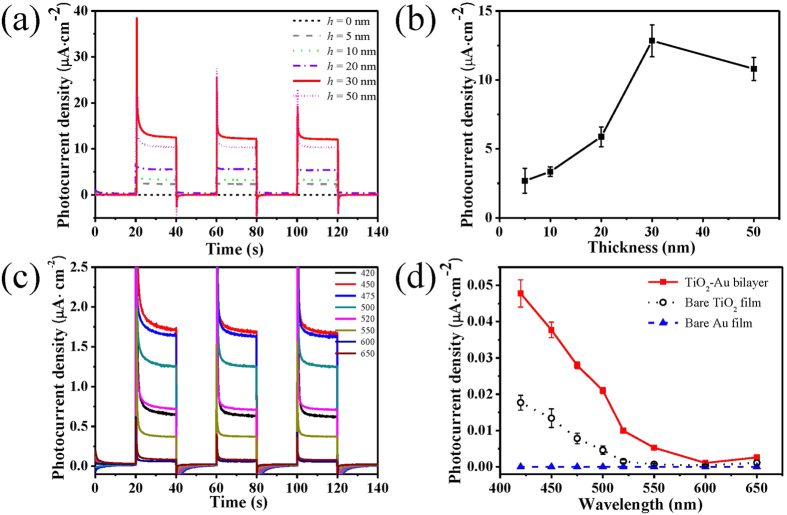
(**a**) *I-t* plots and (**b**) photocurrent densities of the TiO_2_-Au bilayer samples with the TiO_2_ thicknesses of 0, 5, 10, 20, 30 and 50 nm, here the sample of 0 nm represents the rough TiO_2_ film itself; (**c**) *I-t* plots under the irradiation of different monochromatic wavelengths; and (**d**) action spectra (i.e., photocurrent versus wavelength) of the TiO_2_-Au bilayer (red line) and its constituent layers – the bare TiO_2_ film (black line) and the bare Au film (blue line), here the TiO_2_ film is always 30 nm thick.

The results of *I-t* measurements are plotted in Fig. 4a for the TiO_2_-Au bilayer samples with different TiO_2_ film thicknesses. It is seen that the photocurrents respond immediately and repeatedly to the turning on and off of light source. Figure 4b plots the measured photocurrent density as a function of the TiO_2_ thickness, showing the maximum 12.4 μA·cm^−2^ at 30 nm. Therefore, the TiO_2_-Au bilayer sample with the 30-nm-thick TiO_2_ film has the highest photocurrent, this sample is named as the “optimal bilayer sample”. To further investigate the wavelength dependence, the optimal bilayer sample is illuminated with a broadband visible light source (*λ* ≥ 400 nm) that delivers a total power of ~300 mW/cm^2^ (see Fig. S3a of Supplementary Information for the calibrated emission spectrum). The light source employs a series of narrow-band optical filters to filter the broadband light into roughly monochromatic light. The source intensity as a function of the wavelength using the optical filters is plotted in Fig. S3b, agreeing with the spectrum of visible light source (see Fig. S3a)^27^. The *I*-*t* plots of the optimal bilayer sample are shown in Fig. 4c for the wavelengths of 420, 450, 475, 500, 520, 550, 600 and 650 nm, respectively. As a supplementary, the *I*-*t* plots of the bare TiO_2_ film and the TiO_2_-AuNP hybrid are also shown in Fig. S3c,d, respectively. For quantitative comparison, the action spectra (i.e., photocurrent density versus light wavelength) are plotted in Fig. 4d for the optimal bilayer sample and its constituent layers–the 30-nm-thick bare TiO_2_ film and the bare Au film. It is seen that the bare Au film has almost no response whereas the optimal bilayer sample has the highest result, showing significant enhancement as compared to the bare TiO_2_ film. For the optimal bilayer sample, the photocurrent density drops quickly when the wavelength goes up to 600 nm and becomes very low afterward. The energy conversion efficiencies of the three samples can also be indicated by the incident photon to current efficiency (IPCE), which are extracted from the measured photocurrents and the incident spectrum. The IPCE of the optimal TiO_2_-Au bilayer sample reveals a clear enhancement as compared to the bare TiO_2_ film and the bare Au film, which is consistent with the action spectra plotted in Fig. 4d.

### Simulation

**Figure 5:**
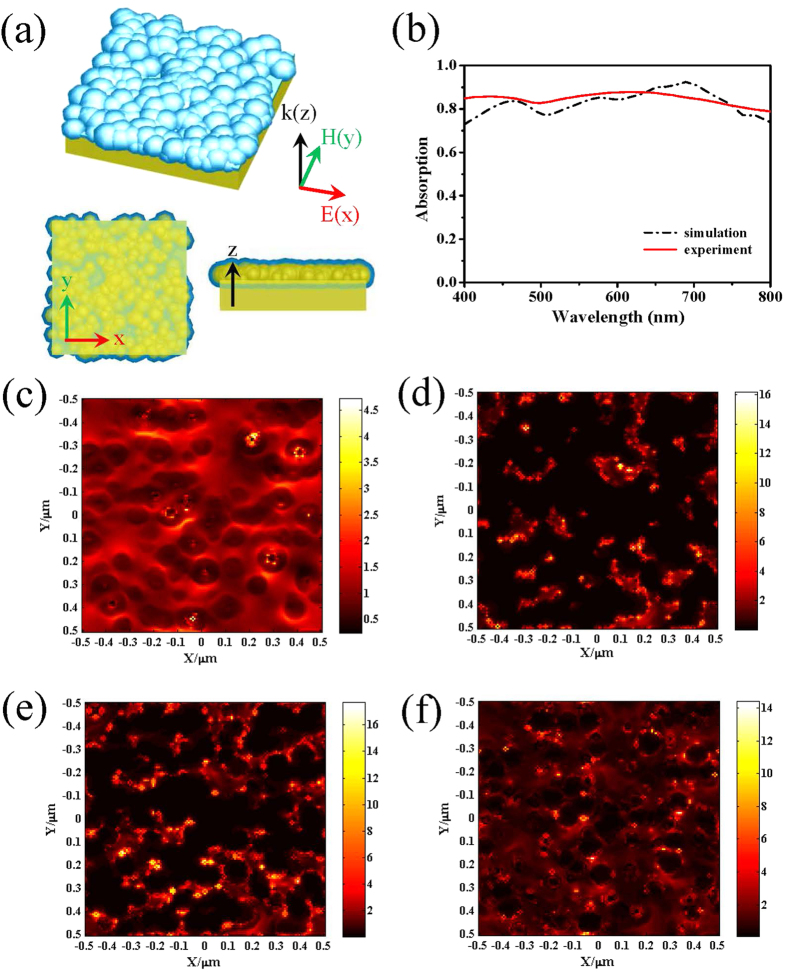
Simulation results. (**a**) Perspective view, top cross-sectional view (XY plane) and side cross-sectional view (XZ plane) of the model for the TiO_2_-Au bilayer sample; (**b**) comparison of the measured absorption spectrum with the calculated one using the FDTD method for the TiO_2_-Au bilayer sample whose TiO_2_ film is 30-nm thick; The electric field distributions on the transverse cross-sections located at (**c**) *Z* = 0 nm, (**d**) *Z* = 30 nm, (**e**) *Z* = 60 nm and (**f**) *Z* = 90 nm, here *Z* = 0 is at the upper surface of the flat Au layer.

Based on the physical structure and materials of the actual TiO_2_-Au bilayer sample, the simulation model is built up as depicted in Fig. 5a, with the Au portion and the TiO_2_ portion denoted by yellow and blue color, respectively. The model consists of three layers, from bottom to top, a flat Au film layer, an Au NPs layer and a TiO_2_ layer. The FTO thin film is not included in the simulation since there is almost no transmission of light through the Au layer. As an approximation, the thickness of the flat Au film layer is 100 nm in the simulation. Considering the roughness of the Au film (see the AFM image in Fig. 3b), the rough surface is represented by randomly-distributed particles^28^. In the simulation, the particles are randomly scattered on the surface in a space of 1 μm × 1 μm × 30 nm, with their diameters varying from 60 to 120 nm. The vertical positions of the particles are adjusted so that the highest particles are always attached to the upper boundary of the flat Au layer while the other particles may be partially embedded into the flat Au layer. The Au nanoparticles can be overlapped with each other, in which some larger domain can be formed occasionally to further account for the randomness of the distribution, the shape and the size. To mimic the TiO_2_ film, the template method is adopted^29^. Each Au NPs is embraced by a shell layer of TiO_2_, whose thickness is equal to the ALD deposition thickness approximately. For the bare area without the Au NPs covered, a flat TiO_2_ film layer with the deposition thickness is also added above the flat Au layer. The meshing order of these Au NPs has higher priority than the TiO_2_ layers to ensure the coverage by TiO_2_ at only the outer boundaries. The transverse dimension of the model structure is 1 μm × 1 μm and the number of Au NPs is 320 in the simulation. With the random positioning, the number of Au NPs is essential for simulating the surface roughness of the Au film, and a relatively small number generally implies a larger surface roughness. In this way, the roughness of Au surface of the real TiO_2_-Au bilayer can be well represented.

Regarding the boundary condition, Perfectly Matched Layer (PML) is used for both the top and bottom boundaries, while periodic condition is employed for the other side boundaries. The periodic condition is a good approximation as the model’s transverse dimension is large as compared to the particle size to support the randomness of distribution. The 4^th^ level auto non-uniform meshing is applied to balance the simulation accuracy and the computation load. The S parameter analysis group is implemented for the absorption analysis, in which a plane source is used and the far-field measurement is conducted. In the simulations, the average thickness of the TiO_2_ layer is varied from 5 to 50 nm. The absorption spectrum of the absorber is recorded for each thickness, within the wavelength range of 400–800 nm.

The simulated absorption spectrum (black line) for the TiO_2_-Au bilayer sample with the 30-nm TiO_2_ film resembles approximately the measured spectrum (red line) as shown in Fig. 5b. The maximum absorption is about 90% near 650 nm, and a shallow dip occurs near 500 nm. As for the various values of TiO_2_ thickness, including 5, 10, 20, 30, and 50 nm, the absorption spectra of the absorbers are presented in Fig. S4, which coincide with the experimental results in Fig. 3d. The optimal absorption occurs at the thickness of 30 nm. To further understand the optical property of the 30-nm TiO_2_-Au bilayer, the electric field distributions are also simulated in some transverse cross-sections. The position *Z* of the cross-section is defined using the vertical displacement from the upper surface of the flat Au layer. The electric field distributions for cross-sections at *Z* = 0, 30, 60, and 90 nm are depicted in Fig. 5c–f, respectively. It can be seen that some hot spots occur near the sites of the particles, indicating the LSPR effect and the plasmonic coupling effect. These effects play a central role in the overall enhancement of absorption. For the broadband absorption, the shell layer and the flat layer of TiO_2_ can be regarded as the effective index media for index matching. The TiO_2_ layer also modifies the coupling between the Au NPs, which can be used to tailor the absorption in the visible light band.

## Discussions

### Mechanism of photocurrent generation

**Figure 6:**
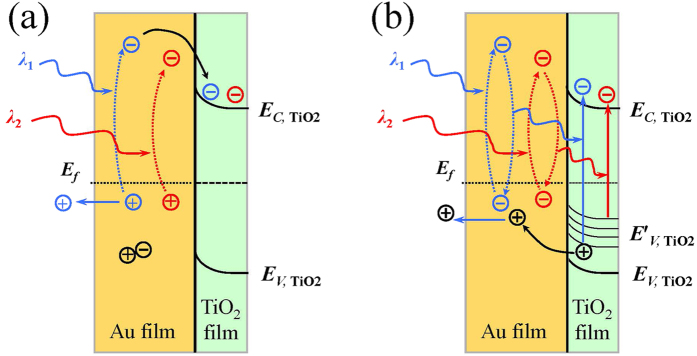
Mechanisms of the photocurrent generation in the TiO_2_-Au bilayer. (**a**) Hot electron injection, for which the rough Au film absorbs photons of different wavelengths to generate hot electrons, whose potential levels of excited states are high enough to overcome the potential barrier to be injected into the TiO_2_ layer. (**b**) Plasmonic resonance energy transfer, for which the plasmonic resonance of Au nanostructures generates intense electric fields in the TiO_2_ layer and excites the electrons of TiO_2_ to the conduction band. For the energy’s point of view, the photon energy excites the Au electrons, which falls back and transfers the energy to the TiO_2_ electrons via the intense electric field near the plasmonic hot spots.

For effective generation of photocurrent, the optical absorption is only the first step. As mentioned above, the enhancement of optical absorption is due to the LSPR effect and the plasmonic coupling of the random Au nanostructures in the rough Au film^5^,^30^,^31^,^32^. The next step to the photocurrent generation relies on the excitation, separation and transfer of charge carriers. Figure 6 depicts the two mechanisms of the photocurrent generation in the TiO_2_-Au bilayer: *hot electron injection* (HEI) and *plasmonic resonance energy transfer* (PRET). Both mechanisms are widely used for the plasmonic enhancement of photocurrent in literature^2^,^5^,^6^,^17^,^18^,^27^. The HEI is illustrated in Fig. 6a, the light incident on the sample excites the plasmonic resonance of electrons in the Au nanostructures, part of these plasmons decays into hot electrons, which are then fed into the conduction band of TiO_2_. This enables the creation of active electrons in the TiO_2_ nanoparticles even in the absence of direct light absorption by TiO_2_^33^,^34^. As the rough Au film of the TiO_2_-Au bilayer enables the plasmons at different wavelengths, hot electrons of different energies may be excited and injected to the TiO_2_ conduction band. In contrast, the PRET goes through a different way as shown in Fig. 6b. The plasmonically excited electrons in the Au film do not migrate into the TiO_2_ film, instead they can transfer the energy to the valence-band electrons of TiO_2_ film to excite them to the conduction band of TiO_2_. This energy transfer process can be enabled by the intense electric field of plasmonic resonance. More specifically, the incident photons excite the collective oscillation of the electrons in Au nanostructures, and thus generate intense oscillating electric fields nearby the Au surface. The region with intense electric field is often called hot spot. The hot spots can penetrate into the TiO_2_ film (typically by a distance of ~10 nm) and thus the oscillating electric fields can excite the TiO_2_ electrons. The HEI and the PRET have different requirements. The former needs the Au nanostructures to be directly in physical contact with the TiO_2_ for hot electron transfer and also the photon energy to be high enough to overcome the potential barrier at the Au/TiO_2_ interface, which has a barrier height of about ~1 eV. The latter requires the overlapping of the absorption spectra of the Au nanostructure and the TiO_2_ film, and needs also the extension of the hot spot into the TiO_2_ film. Fortunately, all of these conditions are satisfied in our TiO_2_-Au bilayer. For example, the rough Au surface is directly covered by the TiO_2_ film, and thus the direct electron transfer is enabled and the hot spots can cover part of the TiO_2_ film near the rough Au surface; over the strong absorption band of 400–800 nm of the TiO_2_-Au bilayer (see Fig. 1b), the corresponding photons energy is in the range of 3.1–1.55 eV, sufficiently higher than the potential barrier ~1 eV; in addition, the TiO_2_ film has certain absorption over 400–700 nm (see Fig. 1b) due to the defects in the TiO_2_ film whereas the rough Au film itself has flat absorption over 400–800 nm (see Fig. S2e), enabling the overlapping of the absorption spectra. Based on these, we attribute to both the HEI and the PRET for the photocurrent enhancement.

For the influence of the TiO_2_ film thickness on the photocurrent of the TiO_2_-Au bilayer structure, the TiO_2_ film serves three functions. First, the TiO_2_ film is a dielectric layer and acts as the index matching layer and assists the optical coupling of the incident light into the rough Au film^18^. Thicker or thinner TiO_2_ film would deteriorate the effects of index matching and optical coupling. As a result, there is an optimal TiO_2_ thickness for optical coupling. Second, the ALD-deposited and thermally-annealed TiO_2_ film has more defects and causes additional energy states inside the TiO_2_ bandgap, enabling to absorb visible light. Since the defects and the associated energy states vary with the TiO_2_ thickness, the absorption of visible light is changed. Third, the TiO_2_ film of the TiO_2_-Au bilayer provides an electron receptor and also an diffusion layer for the transport of the hot electrons to the TiO_2_/solution interface for photochemical reactions^35^,^36^. Thin TiO_2_ film causes low volume to receive electrons but enables fast transport of electrons due to the short diffusion length, whereas thick TiO_2_ film benefits the electron reception but impairs the electron transport due to the long diffusion length. The combined effect of the optical coupling, the defect-induced visible absorption and the electron reception/transport results in the existence of an optimal thickness of TiO_2_ film, which happens to be 30 nm in this work. This is the reason that the TiO_2_-Au bilayer yields the highest photocurrent when the TiO_2_ film is 30 nm thick, as shown in Fig. 4b. As a consequence, the optimal efficiency of photocurrent generation is achieved when the TiO_2_–Au bilayer sample has the 30-nm-thick TiO_2_ film.

In conclusion, we have examined the rough Au film to sensitize TiO_2_ to visible light over 400–800 nm. Burying the rough Au film under the TiO_2_ film forms the TiO_2_-Au bilayer and induces the broadband optical absorption and the significant enhancement of photocurrent as compared to the other two types of control samples, the TiO_2_-AuNP hybrid and the bare TiO_2_ film. Such enhancement is ascribed to the LSPR and coupling effects of the random Au nanostructures in the rough Au film for the strong visible absorption and to also the Schottky junction in the Au/TiO_2_ interface for the separation of photogenerated electrons and holes. Besides, the optimal thickness of TiO_2_ film for the TiO_2_-Au bilayer is found to be 30 nm. The superior optical and photoelectrochemical properties of the TiO_2_-Au bilayer demonstrate its great potential as the photoelectrode for future environmental and energy technologies. Moreover, this new photoelectrode possesses the merits of high absorption efficiency, broadband absorption of visible light, low production cost and easy fabrication process, and brings new insight into the design and preparation of advanced visible-light responsive photocatalytic materials.

## Methods

### Preparation of rough Au films, Au nanoparticles and TiO_2_ films

Deposition of rough Au film on the FTO glass is performed in a magnetron sputtering system that is equipped with three cathodes in a pure Ar atmosphere at 6 × 10^−3 ^Pa. Before deposition, the FTO glass with a size of 10 × 10 × 2 mm^3^ is rinsed successively with acetone and methanol in an ultrasonic bath for 10 min, and then dried with a pure nitrogen flow. The separation distance between the target and the substrate is maintained at 10 cm throughout the deposition, while the substrate speed is set at 6 rpm. For the formation of rough Au films, a thin Cr film is first deposited on the FTO as an adhesion layer, and a 100 nm-thick Au film is then deposited by the sputtering method. For the fabrication of Au NPs, a thin Au film of about 8 nm thick is deposited on the FTO glass, which is transformed into Au NPs after the annealing process at 500 °C for 1 h in a furnace. Among various preparation methods of Au NPs, this physical sputtering method is simple, low cost, and easy to fabricate diverse composite films with uniformly distributed metal particles^37^,^38^.

The TiO_2_ film is deposited onto three different substrates, including the Au film, the Au NPs and the FTO glass slide, respectively, using a commercial atomic laser deposition (ALD) reactor (Cambridge NanoTech, G2 Savannah). Correspondingly, three types of samples, the TiO_2_-Au bilayer, the TiO_2_-AuNP hybrid and the bare TiO_2_ film, are obtained as shown in Fig. 1a. The ALD procedure involves an alternating exposure of Tetrakis dinethylamido titanium (TDMAT), Ti(NMe_2_)_4_ and deionized water vapor as the precursor carriers at a process temperature of 100 °C with N_2_, and then the purge gas at a pressure of 90 sccm. Both the pulse time and the purge time for the precursors are 2 s. The deposition rate of TiO_2_ is estimated to be 0.55 Å per cycle. The thickness of TiO_2_ film is tuned by controlling the deposition time. After the ALD deposition, the TiO_2_ films are annealed at 500 °C for 1 h. To study the influence of TiO_2_ thickness, TiO_2_ films with a series of thicknesses (i.e., 5, 10, 20, 30 and 50 nm) are deposited on the 30-nm-thick Au film to obtain five samples of the TiO_2_-Au bilayer.

### Characterization of bilayer structures

The morphology and the particle size of the samples, including the Au film, the Au NPs and the TiO_2_ film, are observed by scanning electron microscope (SEM, JEOL Model JSM-6490). The surface morphology and the height information of the Au film are also obtained by atomic force microscope (AFM, Veeco Nanoscope V). The X-ray diffraction (XRD) pattern is recorded using a X-ray diffractometer (Rigaku SmartLab). The TiO_2_ films with different thicknesses on the Au film are recorded using the Cu K_α_ radiation and the scanning speed of 5°/min with the 2*θ* range from 10 to 80°.

### Optical and photoelectrochemical measurements

The optical reflection and transmission spectra of the TiO_2_-Au bilayer, the TiO_2_-AuNP hybrid and the bare TiO_2_ film are recorded over a wavelength range of 400–800 nm using a UV-Vis spectrophotometer (Shimadzu, UV-2550). Reflection measurements are performed with an integrating sphere that uses a BaSO_4_ plate as the reference. This means that both the direct reflected light and the diffuse scattering light are included in the reflection measurement. The photocatalytic reaction and the IPCE action spectra are measured with an electrochemical workstation (CH Instruments, CHI 660E) in a standard three-electrode configuration. To obtain the IPCE action spectra, band-pass filters with a bandwidth less than 15 nm full-width at half-maximum (FWHM) are used. The TiO_2_-Au bilayer, the TiO_2_-AuNP hybrid and the bare TiO_2_ film are used as the working electrode, respectively. A Pt wire electrode is used as the counter electrode and a saturated calomel electrode (SCE) as the reference electrode. Besides, KOH aqueous solution (1 M) is used as the electrolyte. The working electrode is illuminated over an area of about 1 cm^2^ under a simulated solar source (AM 1.5 G, 300 mW/cm^2^) with a UV-cutoff filter to obtain visible light with λ ≥ 400 nm.

### Simulation

A commercial FDTD software (Lumerical FDTD Solutions) is implemented to conduct all the optical simulations. A 3D simulation model is built up according to the physical structure, the material, and the fabrication process of the actual samples.

## Additional Information

**How to cite this article**: Tan, F. *et al*. Rough gold films as broadband absorbers for plasmonic enhancement of TiO_2_ photocurrent over 400–800 nm. *Sci. Rep.* **6**, 33049; doi: 10.1038/srep33049 (2016).

## Supplementary Material

Supplementary Information
