# Supplementary material for: Rough gold films as broadband absorbers for plasmonic enhancement of TiO2 photocurrent over 400–800 nm
Source: Sci Rep. 2016 Sep 9;6:33049. doi: 10.1038/srep33049 (PMC5016800; doi:10.1038/srep33049)
Supplement: Supplementary Information [file srep33049-s1.doc]

**Supplementary Information**

**Rough gold films as broadband absorbers for plasmonic enhancement of TiO_2_ photocurrent over 400 – 800 nm**

Furui Tan^1,2^, Tenghao Li^1^, Ning Wang^1,2^, Sin Ki Lai^1^, Chi Chung Tsoi^1^, Weixing Yu^3^, Xuming Zhang^1,2^,*

*^1^ Department of Applied Physics, The Hong Kong Polytechnic University, Hong Kong, P. R. China.*

*^2^ The Hong Kong Polytechnic University Shenzhen Research Institute, Shenzhen, P. R. China.*

*^3^ Key Laboratory of Spectral Imaging Technology, Xi’an Institute of Optics and Precision Mechanics, Chinese Academy of Sciences, Xi’an, P. R. China.*

**Corresponding author. Tel.:+852-4003258; Fax.: +852-23337629.*

*E-mail:* *apzhang@polyu.edu.hk*

**S1. Scanning electron micrographs of different films**


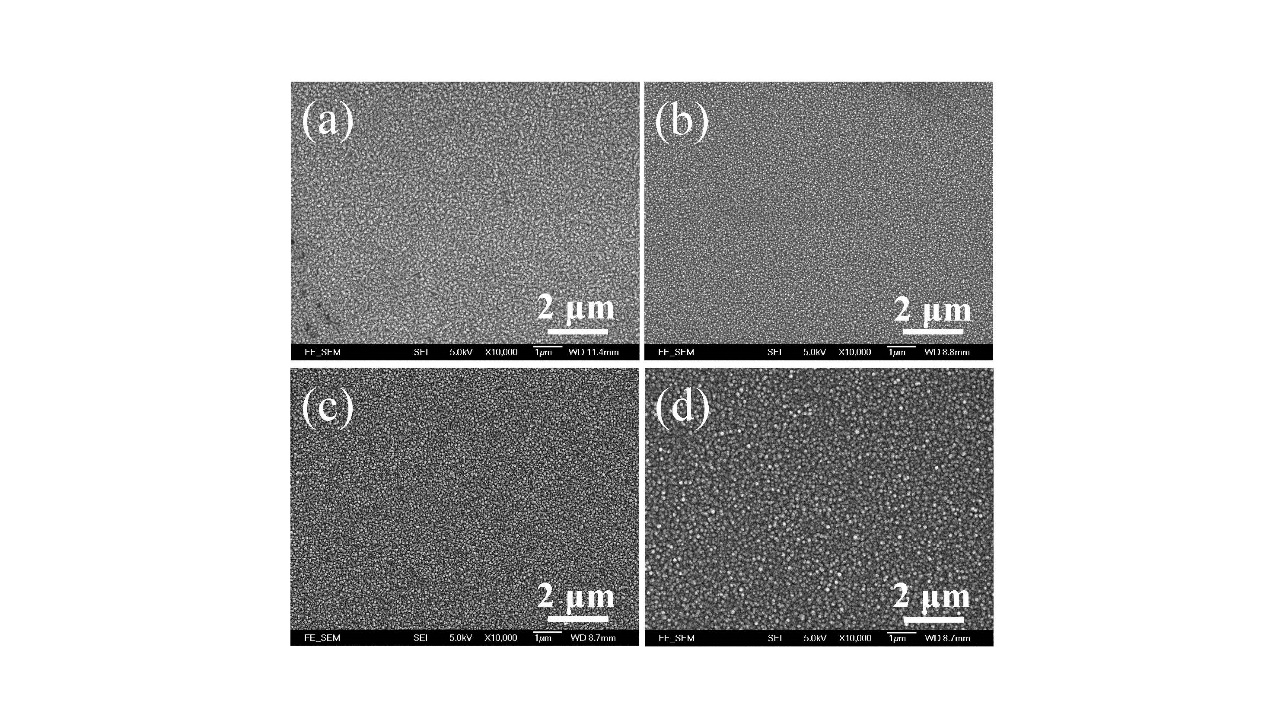


**Figure S1.** SEM surface morphologies of different layers. (a) FTO glass; (b) Au NPs on the FTO glass, deposited by the sputtering process; (c) rough Au film on the FTO glass; (d) TiO_2_ film deposited by the ALD method.

**S2. Reflection and transmission spectra of different samples**


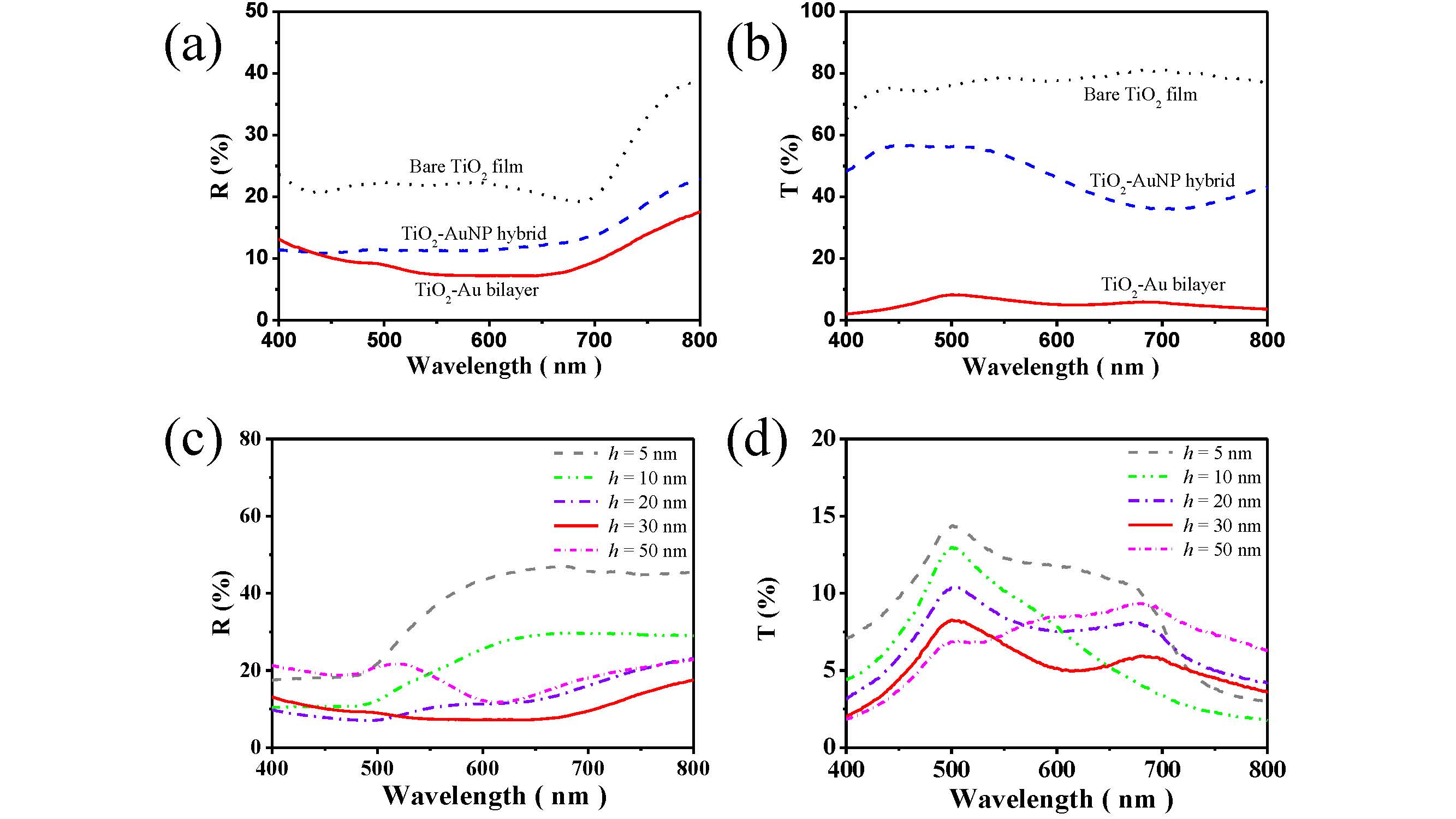


**
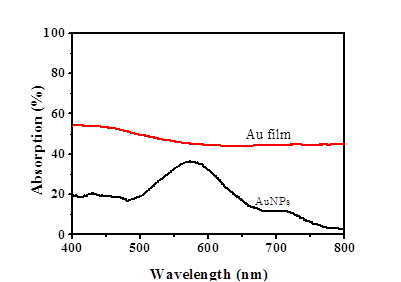
**

(e)

**Figure S2.** (a) Reflection spectra and (b) transmission spectra of the bare TiO_2_ film (black dashed lines), the TiO_2_-AuNP hybrid (blue dotted lines) and the TiO_2_-Au bilayer (red solid lines), respectively; (c) reflection spectra; (d) transmission spectra of the TiO_2_-Au bilayer samples with the TiO_2_ thicknesses of 5, 10, 20, 30 and 50 nm; and (e) absorption spectra of the rough Au film itself and the bare Au NPs. It is noted that the ranges of *y* axis are different, i.e., 0 – 50% in (a), 0 – 100% in (b), 0 – 80% in (c), 0 – 20% in (d) and 0 – 100% in (d). Here the reflection measurements are performed with an integrating sphere that uses a BaSO_4_ plate as the reference. This means that both the direct reflected light and the diffuse scattering light are included in the reflection measurement.

**S3. Detailed experimental data**


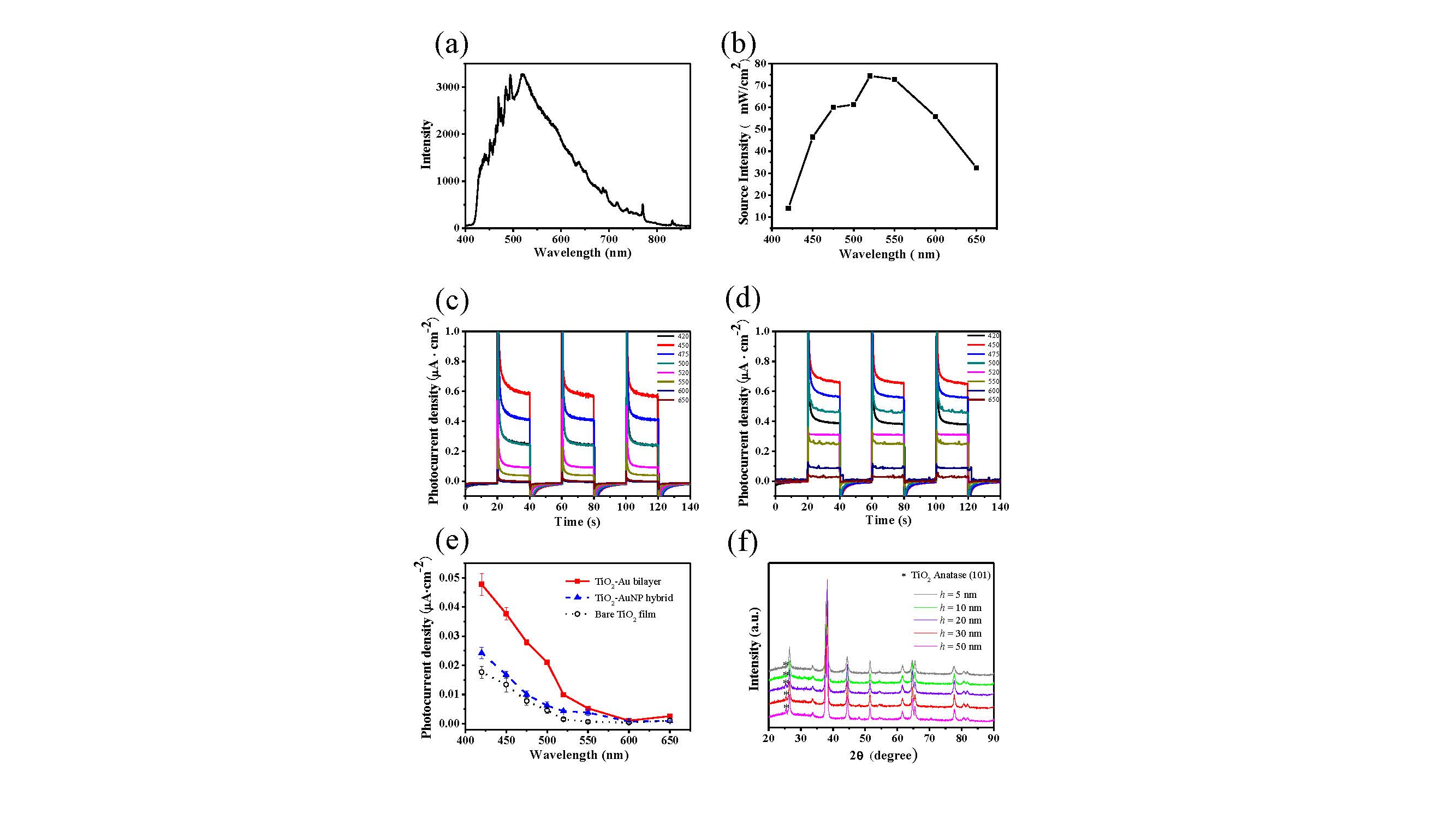


**Figure S3.** (a) Calibrated emission spectrum of the light source after the UV-cutoff filter (> 400 nm), the overall intensity is ~ 300 mW/cm^2^ at the photoelectrode surface. (b) Calibrated optical intensities of the light source at different wavelengths when the narrow-band optical filters are used to get monochromatic light for the measurement of action spectrum (see Fig. 4(d)). *I-t* plots of (c) the bare TiO_2_ film and (d) the TiO_2_-AuNP hybrid under monochromatic lights of 420, 450, 475, 500, 520, 550, 600 and 650 nm, respectively. Here the TiO_2_ film is always 30 nm thick. (e) Action spectra (i.e., photocurrent versus light wavelength) of the TiO_2_-Au bilayer (red solid line), the TiO_2_-AuNP hybrid (blue dashed line) and the bare TiO_2_ film (black dotted line), in these three samples the TiO_2_ layers are all 30 nm thick. (f) XRD plots of the TiO_2_-Au bilayer samples with the TiO_2_ film thicknesses of 5, 10, 20, 30 and 50 nm. For all the samples, the diffraction peak appears at 2*θ* = 25.3°, corresponding to the (101) orientation of anatase phase, while the other diffraction peaks are very weak. This indicates that the crystallographic phases of these TiO_2_ films are all anatase-type. Moreover, the XRD intensity of these TiO_2_ films goes up with the increase of the TiO_2_ thickness.

**S4. Simulated absorption spectra**


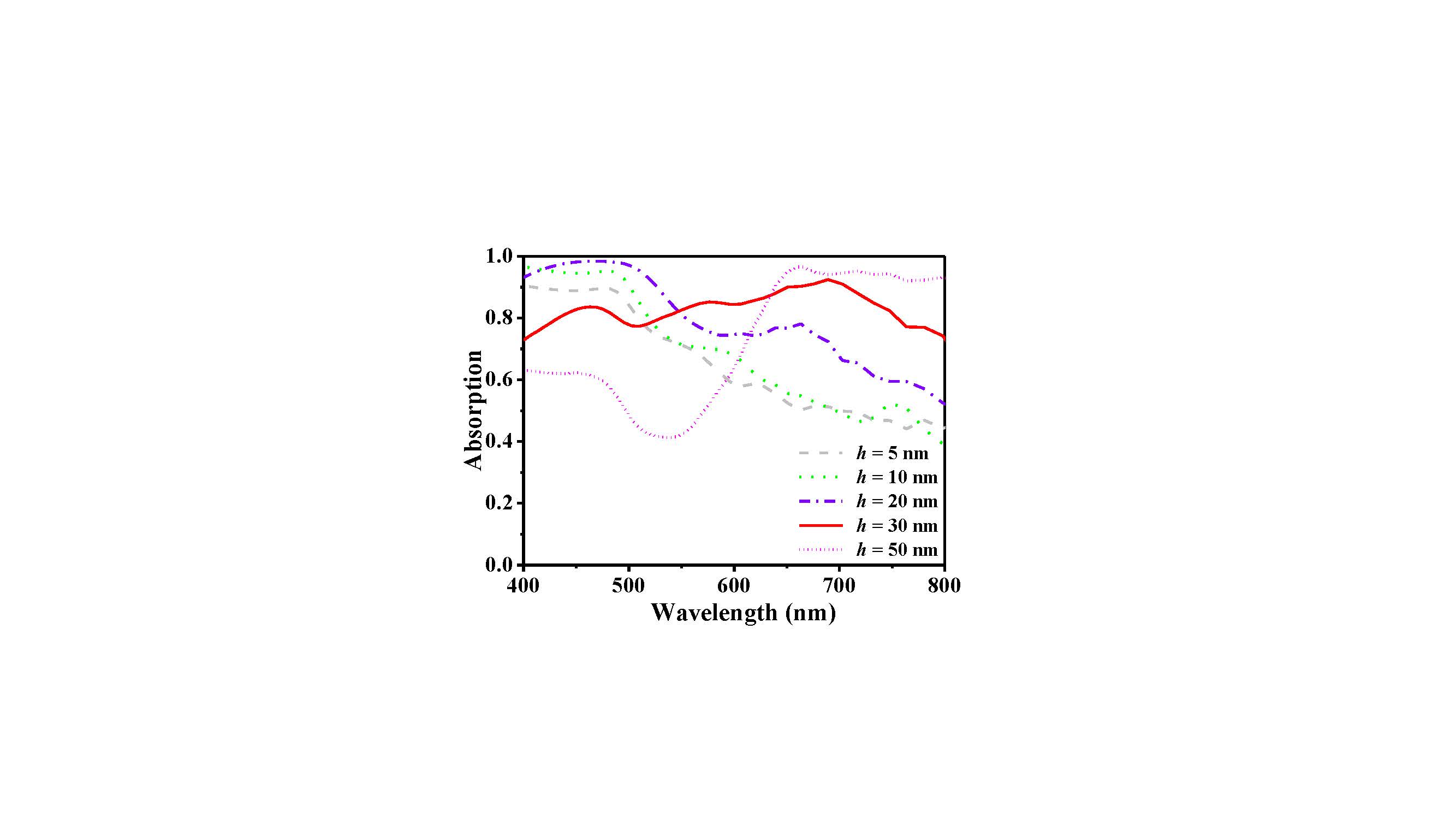


**Figure S4.** Simulated results of the absorption spectra of the TiO_2_-Au bilayer samples. The thickness of TiO_2_ film varies from 5, 10, 20, 30 to 50 nm.

**S5. Parameters of gold and TiO_2_ used in simulation**

The refractive indices of Au are simulated using the built-in data of FDTD Solution software. The category labeled as CRC is chosen, which provides the model by fitting the experimental data in CRC Handbook of Chemistry & Physics^1^. The refractive indices of TiO_2_ are simulated using the model presented in Ref. 2, with the dispersion formula as *n*^2^ = 5.913 + 0.2441/(*λ*^2^ − 0.0803)^2^. The wavelength is denoted as *λ* in the dispersion formula with the unit as micron.

**Reference**

1. D. R. Lide, *Default optical material database.* Available at: <https://kb.lumerical.com/en/materials_default_optical_database.html> (Accessed: 18 May 2016)
2. J. R. Devore, Refractive Indices of Rutile and Sphalerite. *J. Opt. Soc. Am.* **1951**, *41* (6), 416.
